# Immunotherapy to improve cognition and reduce pathological species in an Alzheimer’s disease mouse model

**DOI:** 10.1186/s13195-018-0384-9

**Published:** 2018-06-18

**Authors:** Krystal Herline, Frances Prelli, Pankaj Mehta, Claire MacMurray, Fernando Goñi, Thomas Wisniewski

**Affiliations:** 1 Center for Cognitive Neurology and Department of Neurology, New York University School of Medicine, Alexandria, ERSP Rm 802, 450 East 29th Street, New York, NY USA; 2 Department of Immunology, New York State Institute for Basic Research in Developmental Disabilities, Staten Island, USA; 3 Quest University Canada, Squamish, BC Canada; 4 Departments of Pathology and Psychiatry, New York University School of Medicine, New York, NY USA

**Keywords:** Alzheimer’s disease, Amyloid-beta (Aβ), Phosphorylated tau, Oligomers, Passive immunization, Monoclonal antibody, 3xTg-AD, Mouse model

## Abstract

**Background:**

Alzheimer’s disease (AD) is characterized by physiologically endogenous proteins amyloid beta (Aβ) and tau undergoing a conformational change and accumulating as soluble oligomers and insoluble aggregates. Tau and Aβ soluble oligomers, which contain extensive β-sheet secondary structure, are thought to be the most toxic forms. The objective of this study was to determine the ability of TWF9, an anti-β-sheet conformation antibody (aβComAb), to selectively recognize pathological Aβ and phosphorylated tau in AD human tissue compared with cognitively normal age-matched controls and to improve the performance of old 3xTg-AD mice with advanced pathology in behavioral testing after acute treatment with TWF9.

**Methods:**

In this study, we used immunohistochemistry, immunoprecipitation, and enzyme-linked immunosorbent assay (ELISA) to characterize TWF9 specificity. We further assessed cognitive performance in old (18–22 months) 3xTg-AD mice using both a Barnes maze and novel object recognition after intraperitoneal administration of TWF9 (4 mg/kg) biweekly for 2 weeks before the start of behavioral testing. Injections continued for the duration of the behavioral testing, which lasted 2 weeks.

**Results:**

Histological analysis of TWF9 in formalin-fixed paraffin-embedded human control and AD (ABC score: A3B3C3) brain tissue revealed preferential cytoplasmic immunoreactivity in neurons in the AD tissue compared with controls (*p* < 0.05). Furthermore, ELISA using oligomeric and monomeric Aβ showed a preferential affinity for oligomeric Aβ. Immunoprecipitation studies showed that TWF9 extracted both phosphorylated tau (*p* < 0.01) and Aβ (*p* < 0.01) from fresh frozen brain tissues. Results show that treated old 3xTg-AD mice have an enhanced novel object recognition memory (*p* < 0.01) and Barnes maze performance (*p* = 0.05) compared with control animals. Overall plaque burden, neurofibrillary tangles, microgliosis, and astrocytosis remained unchanged. Soluble phosphorylated tau was significantly reduced in TWF9-treated mice (*p* < 0.05), and there was a trend for a reduction in soluble Aβ levels in the brain homogenates of female 3xTg-AD mice (*p* = 0.06).

**Conclusions:**

This study shows that acute treatment with an aβComAb can effectively improve performance in behavioral testing without reduction of amyloid plaque burden, and that peripherally administered IgG can affect levels of pathological species in the brain.

## Background

Alzheimer’s disease (AD) is a devastating neurodegenerative disease neuropathologically characterized by two physiological endogenous proteins, amyloid beta (Aβ) and tau, undergoing conformational changes and accumulating as soluble oligomers and insoluble aggregates [1–9]. These insoluble aggregates, neurofibrillary tangles (NFTs) and amyloid plaques, are now thought to be relatively inert, whereas soluble Aβ and tau oligomers are thought to be the most toxic forms [10–15]. Although there are various oligomeric species, soluble tau and Aβ oligomers are thought to share a pathological predominately β-sheet conformation [14–20].

Currently there is no effective disease-modifying therapy for AD. One reason for this is that some therapeutic approaches have targeted relevant physiological forms and thus caused toxicity, without specifically targeting the most toxic oligomeric species [18, 21]. Secondly, trials have failed to simultaneously target both phosphorylated tau and Aβ, and have only targeted them in isolation [16, 18, 22]. Thirdly, the direct targeting of fibrillar vessel amyloid deposits has been associated with complications such as amyloid-related imaging abnormalities (ARIA) [23–26]. Recent reports have shown new immunotherapeutic approaches that might overcome these shortcomings [18, 19, 22, 27].

Our group has recently developed anti-β-sheet conformation monoclonal antibodies (aβComAb) that were raised to a nonfibrillogenic and non-self-oligomeric antigen with a repetitive β-sheet secondary structure. The monoclonal antibodies recognize multiple misfolded protein/peptides of various neurodegenerative diseases [22]. One of these monoclonals, aβComAb IgMκ GW-23B7, in a passive immunization preclinical trial in old 3xTg-AD mice consisting of seven injections over a 2-month period was able to reverse cognitive deficits in old mice, as well as reduce Aβ and tau oligomers levels [27]. This study suggests that IgMκ GW-23B7 may be a valuable biochemical tool for immunotherapeutics. However, as of date, all Food and Drug Administration (FDA) approved passive immunotherapies are restricted to IgG molecules of different subclasses, but none are monoclonal IgM antibodies. Moreover, IgMs are stronger activators of complement than IgGs [28]. The mode of action of complement inside the brain has been known to exacerbate neurodegeneration, thus making IgGs a better therapeutic option than an IgM [29]. Therefore, we engineered an IgG2aκ antibody using the whole light chain and the heavy chain variable region from the parent aβComAb IgMκ GW-23B7.

In this study we wanted to determine the ability of our IgG anti-β-sheet conformation monoclonal antibody, aβComAb TWF9, to reverse cognitive deficits in old 3xTg-AD mice with a short treatment interval. We hypothesized that soluble levels of pathological tau and/or Aβ would be reduced but insoluble species, such as amyloid plaques and NFT pathology, would be unaffected.

## Methods

### Patients and clinical evaluations

For human studies, formalin-fixed paraffin-embedded (FFPE) human brain tissue and fresh frozen brains from patients with AD, mild cognitive impairment (MCI) patients with varying Aβ and tau pathology and with cognitive deficits insufficient to be classified as dementia with a Global Dementia Scale (GDS) score of 3, and nondemented, age-matched control cases also with varying Aβ and tau pathology were used (Tables 1 and 2) [30]. All cases were randomly chosen from the NYU Alzheimer’s Disease Center based on tissue availability. Individual patient information including gender, age, neuropathological assessment/classification, and ABC score is included in Tables 1 and 2 [31].

**Table 1:** Formalin-fixed paraffin-embedded cases used for fluorescent immunohistochemistry

Case	GDS	Sex	Age (years)	ABC	Neuropathological diagnosis
Cognitively normal control 1	≤2	M	63	A1,B1,C0	Atherosclerosis
Cognitively normal control 2	≤2	F	71	A0,B1,C0	Atherosclerosis
Cognitively normal control 3	≤2	M	65	A1,B0,C0	Arteriosclerosis
Cognitively normal control 4	≤2	M	69	A0,B0,C0	Normal
Cognitively normal control 5	≤2	M	60	A0,B0,C0	Atherosclerosis
Cognitively normal control 6	2	F	84	A0,B1,C0	Normal
Cognitively normal control 7	2	M	84	A1,B2,C1	ADRC
MCI 1	3	F	97	A1,B2,C1	ADRC
MCI 2	3	M	95	A0,B1,C0	Small vessel disease
MCI 3	3	F	88	A2,B2,C2	ADRC/small vessel disease
MCI 4	3	F	88	A2,B2,C2	ADRC/peripheral vascular disease
MCI 5	3	M	84	A2,B2,C2	Lacunar infarct
MCI 6	3	M	85	A2,B2,C2	CAA
MCI 7	3	F	90	A0,B2,C0	Infarcts/CAA
MCI 8	3	F	83	A2,B2,C2	ADRC/ischemic damage
AD 1	≥5	F	83	A3,B3,C3	AD
AD 2	≥5	F	80	A3,B3,C3	AD
AD 3	≥5	F	80	A3,B3,C3	AD
AD 4	≥5	F	100	A3,B3,C3	AD
AD 5	≥5	M	81	A3,B3,C3	AD
AD 6	≥5	M	84	A3,B3,C3	AD
AD 7	≥5	M	74	A3,B3,C3	AD
AD 8	≥5	M	79	A3,B3,C3	AD
AD 9	≥5	F	91	A3,B3,C3	AD
AD 10	≥5	M	69	A3,B3,C3	AD
AD 11	≥5	M	75	A3,B3,C3	AD
AD 12	≥5	F	88	A3,B3,C3	AD
AD 13	≥5	M	66	A2,B2,C2	AD/CAA

ABC score and neuropathological diagnosis was determined as previously published [31] and reported by a board-certified neuropathologist

*AD* Alzheimer’s disease, *ADRC* Alzheimer’s disease-related changes, *CAA* cerebral amyloid angiopathy, *F* female, *GDS* Global Dementia Scale [30], *M* male, *MCI* mild cognitive impairment

**Table 2:** Fresh frozen cases used for immunoprecipitation

Case	Sex	Age (years)	ABC	Neuropathological diagnosis
Cognitively normal control 1	F	95	A0,B0,C0	lacunar microinfarcts, dystrophic calcification in hippocampus
Cognitively normal control 2	M	79	A1,B1,C1	ADRC/small vessel disease
Cognitively normal control 3	M	90	A1,B1,C1	ADRC
AD1	F	91	A3,B3,C3	AD
AD2	M	76	A3,B3,C3	AD/CAA
AD3	F	83	A3,B3,C3	AD

ABC score and neuropathological diagnosis was determined as previously published [31] and reported by a board-certified neuropathologist

*AD* Alzheimer’s disease, *ADRC* Alzheimer’s disease-related changes, *CAA* cerebral amyloid angiopathy, *F* female, *M* male

### IgG antibody expression and purification

**Fig. 1:**
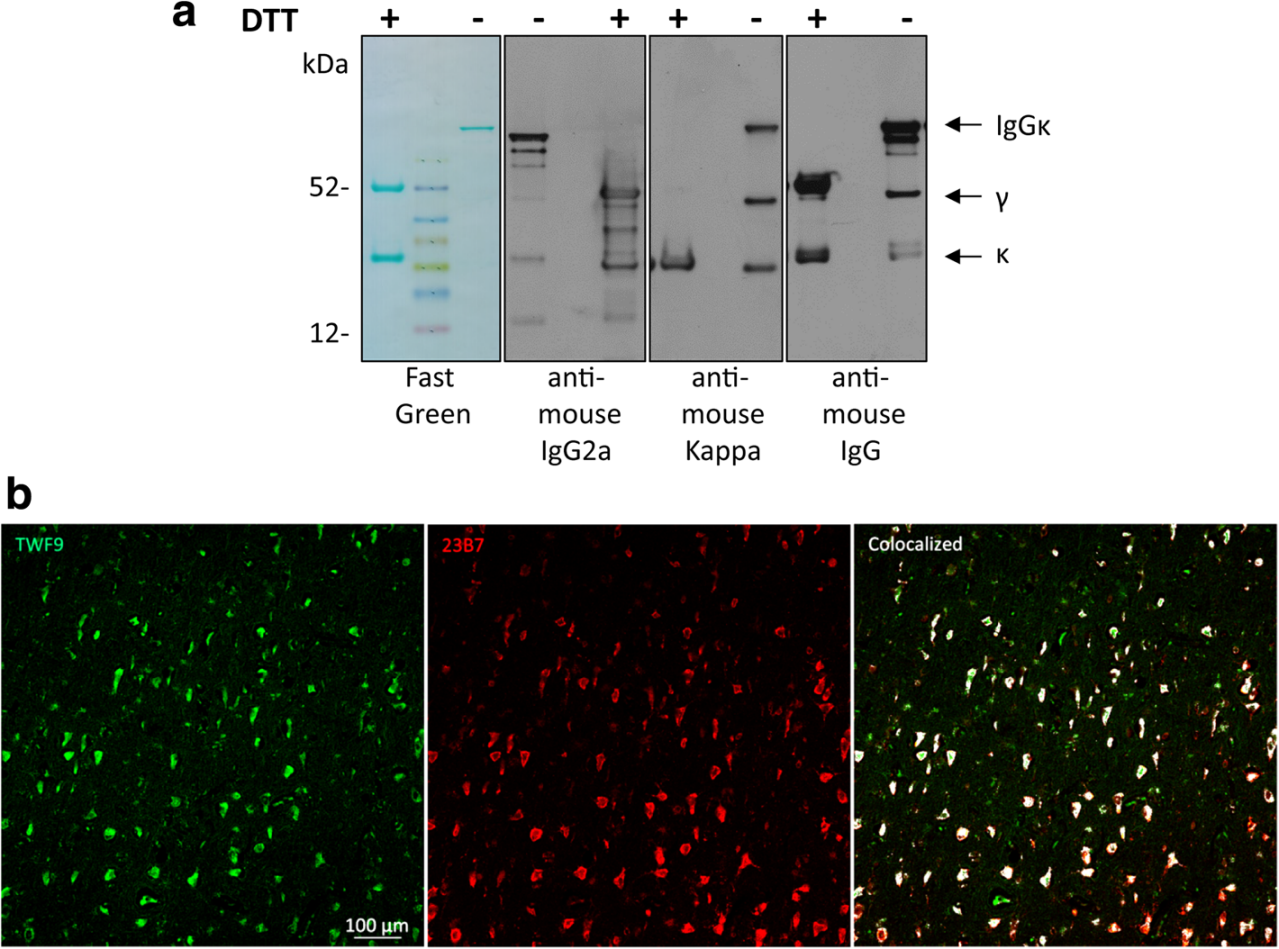
Characterization of TWF9, an anti-β-sheet conformation antibody. **a** TWF9 under reducing (+DTT) and nonreducing (−DTT) conditions. Left panel: fast green reversible protein stain; second panel: anti-mouse gamma 2a specific antibody; third panel: anti-mouse kappa light chain specific antibody; right panel: anti-mouse IgG antibody. **b** Representative image showing staining patterns between GW-23B7 [27] (red) and TWF9 (IgG) (green). Areas of colocalization are shown in white

Genscript (Piscataway, NJ) synthesized the TWF9 antibody using the DNA sequences of the whole kappa chain and the variable region heavy chain of GW-23B7. The DNA sequence for the heavy chain was followed in frame by the DNA sequence for three constant domains and hinge region of a murine ϒ2a immunoglobulin with related tags to facilitate purification. This complete sequence was subcloned into pTT5 vectors for CHO-3E7 cell expression and grown in serum-free FreeStyle™ CHO expression media (Invitrogen, Carlsbad, CA, USA). On day 6, the culture supernatant was collected, centrifuged, and filtered, and then loaded onto MabSelect Columns (GE, cat. no. 17-5199-03). The loading proceeded at 10.0 ml/min, followed by appropriate washing and elution. The pooled fractions of the purified antibody were dialyzed to phosphate-buffered saline (PBS) pH 7.2. The purity and integrity of the TWF9 antibody was analyzed in our laboratory by SDS-PAGE and Western blot (Fig. 1a).

### Fluorescent immunohistochemistry, imaging, and analysis of human and mouse brain tissue

Immunohistochemistry using immunofluorescence of human FFPE tissues has been reported previously [22, 32]. Briefly, 8-μm FFPE human tissue sections were dewaxed and rehydrated through a series of xylene and ethanol incubations. Slides then underwent boiling antigen retrieval in citrate buffer (10 mM sodium citrate, 0.05% Tween-20; pH 6) and washed with PBS containing 0.05% Tween (PBST) three times for 5 min each. Sections were then blocked in a blocking solution (10% normal goat serum (NGS) and 0.2% Triton X-100 in PBS) and incubated overnight with primary antibody in 3% NGS and 0.2% Triton X-100 at 4 °C. The primary antibodies used were TWF9 (1:250) and rabbit tau pSer404 (1:500; Biolegend, CA, USA) or rabbit Aβ42 (rab42; 1:500; a gift from Pankaj Mehta [33]). Prior to the citrate buffer antigen retrieval step for the rab42 stain, sections were first incubated with 88% formic acid for 7 min and then washed four times for 5 min each. The next day, sections were washed and incubated with the appropriate Alexa fluor® 488 and/or Alexa fluor® 647 secondary antibodies (1:500; Jackson ImmunoResearch, West Groove, PA) for 2 h. Afterwards, slides were washed and incubated with Hoechst 33,342 (Sigma) for 10 min to visualize nuclei. Afterwards, sections were washed and coverslipped using PermaFluor™ Aqueous Mounting Medium (Thermo, Waltham, MA).

Immunohistochemistry of 40-μm thick mouse free floating brain tissue has been reported before [34]. Quantification was performed on coronal sections containing the subiculum from all mice in each group. Briefly, sections were washed in PBST, and then incubated for 1 h at room temperature with MOM blocking solution (Vector Laboratories Inc., Burlingame, CA) as described in the kit instructions. After blocking, sections were washed with PBST and incubated with primary antibody overnight in 3% NGS or MOM diluent. The primary antibodies included: GFAP (1:1000; Dako Inc., Carpinteria, CA), IBA1 (1:1000; Wako Chemicals, Richmond, VA), 6E10/4G8 (Covance Research Products, Inc., Denver, PA), PHF1 (1:500; a generous gift of Dr. Peter Davies), MC1 (1:500; from Dr. Peter Davies), and AT8 (1:500; Thermo Scientific). The following day, sections were washed with PBST, incubated with Alexa fluor® secondary antibodies mentioned above at 1:1000 for 2 h, and then washed. Nuclei were stained by incubating the sections in Hoechst 33,342 (Sigma) for 10 min. Afterwards, sections were washed, mounted, and coverslipped.

For the TWF9 specificity assay in FFPE human brain tissue, TWF9 colocalization images, and IBA1 mouse stain, a Zeiss LSM700 confocal microscope at 20× magnification was used to capture three representative Z-stack images through the depth of the 8-μm human sections and the 40-μm mouse sections. The maximum projection image was obtained using ImageJ software and analyzed. All images of a particular stain were collected using the same confocal settings. For the GFAP, tau, and amyloid mouse stains on free floating sections, fluorescent imaging of the whole section was performed at 20× magnification using a NanoZoomer HT2 (Hamamatsu) whole slide scanner using the same settings for all slides. Three to four images containing the subiculum were collected at 10× magnification per case for quantification. Percentage burden was determined by first determining a threshold identifying all pixels with positive labeling for each marker. Then the average threshold value was calculated and used on all sections for each marker. The percentage burden in the region analyzed was calculated as the percentage of positive pixels in the total area of interest.

### Mouse and human brain homogenizing and processing

Flash frozen mouse brain hemispheres were obtained as described previously and homogenized using a PRO 200 Hand-held homogenizer and a 5 mm × 75 mm flat bottom generator probe (Pro Scientific, Monroe, CT) for three cycles of 30 s each at 30,000 rpm, pausing for 30 s between each homogenization cycle [35]. For human brains, small pieces of minced fresh frozen frontal tissue were homogenized using a sonicator (Fisher Scientific Model 60 Sonic Dismembrator) for three cycles for 30 s each, with a 30-s rest in between each cycle. Each subsequent cycle increased in power, with the first cycle at 30% power, the second cycle at 60% power, and the last cycle at 100% power. For brain homogenates used in human and mouse studies, brain tissue was weighed and made to 20% w/v in filtered tissue homogenization buffer (THB) containing 20 mM Tris pH 7.4, 250 mM sucrose, 1 mM ethylenediaminetetraacetic acid (EDTA), and 2.5 mM ethylene glycol-bis(β-aminoethyl ether)-N,N,N′,N′-tetraacetic acid (EGTA). Proteinase inhibitors (1.46 nM pepstatin, 1 mM phenylmethane-sulfonyl-fluoride (PMSF), 1 mM sodium fluoride (NaF), Roche protease inhibitor cocktail (Sigma), and 0.96 mM sodium orthovanadate) were freshly made and added to the THB before homogenization. During the entire process the brains were kept in ice.

The mouse and human 20% brain homogenates were aliquoted (200 μl/each) and stored at −80 °C. An aliquot of 20% brain homogenate was centrifuged for 30 min at a low speed (2200 g) at 4 °C and the upper 90% of supernatant was taken and re-centrifuged for an additional 30 min. The resulting supernatant was used for all human experiments in this study. For mouse brain studies, an aliquot of 20% brain homogenate was centrifuged for 45 min at 14,000 g at 4 °C and the upper 90% of supernatant (S1) was taken. The S1 mouse fraction was used in sandwich enzyme-linked immunosorbent assay (ELISA) experiments to measure soluble Aβ. To measure mouse soluble phosphorylated tau levels via Western blotting, the S1 fraction was subjected to ultracentrifugation at 100,000 g for 1 h at 4 °C. A bicinchoninic acid assay (Pierce, Rockford, IL) of all fractions was performed to ensure equal total protein was used in all studies.

### Immunoprecipitation

Supernatant (150 μg) of total protein from the low-speed centrifugation from various AD and control 20% brain homogenates were incubated with TWF9 and isotype control antibodies; 1.5-mg beads and 25 μg antibody was used per sample. Dynabeads M-270 Epoxy (Invitrogen, USA) protocol and kit buffers were used as per kit instructions. Antibody was crosslinked to beads overnight with rotation at 37 °C. The next day, 150 μg brain homogenate was added to antibody-coupled beads and incubated overnight at 4 °C with rotation. The next day, after a series of washes, the immunoprecipitation product was eluted from the beads and analyzed via Western blot.

### ELISA

For double antibody sandwich ELISA experiments, plates were coated with 100 μl rabbit Aβ42 antibody (2.5 μg/ml) [33] in 50 mM ammonium bicarbonate solution, pH 9.6, overnight at 4 °C. The next day, plates were washed with Tris buffer saline pH 8.3 with 0.05% Tween (TBST). Plates where then blocked with 120 μl Superblock (ThermoFisher) for 2 h at room temperature. The plates were washed and 50 μl of Aβ42 monomers or Aβ42 oligomers (0.5 ng/well) [36] was applied and incubated for 2 h at room temperature. After washing, plates were incubated with 50 μl TWF9, 6E10, or isotype control antibody (Biolegend) at appropriate concentrations for 90 min at room temperature. After washing, 50 μl of goat anti-mouse IgG horseradish peroxidase (1:5000; GE Healthcare, UK) was added and incubated for 1 h. Plates were washed again, and 100 μl of tetramethyl benzidine substrate solution (TMB; Thermo Scientific, Rockford, IL) was added and incubated. The reaction was stopped by adding 100 μl 8 M acetic acid with 1 M sulfuric acid. The optical density (OD) was measured at 450 nm in a plate reader. Results were replicated in independent experiments. The relationship between OD and antibody concentration was determined by a four-parameter logistic log function. Nonlinear curve fitting was performed with GraphPad Prism 7.0 (GraphPad, San Diego, CA).

For direct ELISA experiments to determine the binding affinity (K_D_) value, Immulon 2HB 96-well plates (Thermo, Waltham, MA) were coated per well with 0.2 μg Aβ oligomers, prepared as previously published [36] in 50 mM ammonium bicarbonate solution, pH 9.6, overnight at 4 °C. Blocking, washes, and antibody incubations were followed as described above. The OD was measured at 450 nm in a plate reader. Results were replicated in independent experiments. The K_D_ was determined by saturation binding curve nonlinear regression analysis using one site specific binding data in GraphPad Prism 7.0.

### Electrophoresis and Western blotting of human and mouse brain tissue

Samples were mixed with an equal volume of tricine sample buffer (BioRad, Hercules CA) and then electrophoresed on 12.5% sodium dodecyl sulfate-tris-tricine polyacrylamide gels under reducing conditions and transferred to nitrocellulose membranes via electroblotting as previously described [27]. Membranes were stained with reversible stain Fast Green FCF 0.1% (Fisher Scientific, Waltham, MA) in 25% methanol-10% acetic acid for 1 min, destained with 25% methanol, and then transferred to distilled water to assess equal loading. Membranes were then washed in TBST and blocked for 1 h at room temperature with 5% nonfat dry milk in TBST. Blots were then incubated with PHF1 (1:500), 6E10/4G8 (1:2000), and actin (1:5000; Sigma) overnight at 4 °C. The next day, bound antibodies were detected after 1 h incubation with goat anti-mouse or anti-rabbit IgG HRP (1:3000; GE Healthcare, UK) and the chemiluminescent detection system (Pierce, Rockford, IL) on autoradiography films (MIDSCI, St. Louis, MO).

### Experimental design of the acute study

Two groups of 18- to 22-month-old triple transgenic mice (3xTg-AD; human APP KM670/671NL (Swedish), MAPT P301L, and PSEN1 M146 V) exhibiting amyloid and tau pathologies were used in this acute study [37]. The age of 18–22 months was chosen since we wanted mice with advanced plaque and tangle pathology to determine the effects that removing soluble Aβ and tau would have on cognition. The pathology at this age is more analogous to a patient with symptomatic AD, as it has been well established that even in early AD there is already extensive amyloid plaque and tau pathology [9, 38]. Additionally, it has been reported that different colonies of 3xTg mice can have varying levels of pathology; thus, by waiting until this age range, we would be sure that the mice had advanced AD pathology consistent with our prior published experience using this mouse model [27, 35, 39]. Additionally, C57Bl6 mice were used as nontransgenic (NTg) controls. Although they do not have the same genetic background as the 3xTg-AD mice, we wanted to determine potential therapeutic effects in nontransgenic old mice. Treated Tg animals (*n* = 11) and NTg (*n* = 11) received biweekly intraperitoneal injections of 4 mg/kg TWF9 (100 μg TWF9 for a 25 g mouse) in sterile saline. The 4 mg/kg dosage corresponds to approximately 100 μg/mice/dose. This particular dose was used in our previous GW-23B7 study in aged 3xTg-AD mice where we showed that the concentration of antibody peaked in the brain approximately 24 h after injection [27]. Given this information, we decided that dosing biweekly injections allows for a continual and sustained level of TWF9 antibody in the brain which we believe is necessary to effectively target soluble Aβ and tau central nervous system (CNS) species. Control Tg mice (*n* = 12) and NTg mice (*n* = 9) were treated with saline. For this study, there were 11 transgenic female mice and 12 transgenic male mice. Mice received four injections before the start of sensorimotor testing. On days where injections and testing happened on the same day, injections were performed in the morning and mice were allowed to rest for 3–4 h before the start of the first tests. Animals underwent both motor and behavioral testing. At the conclusion of the studies, animals were given one last injection 24 h before sacrifice. In total, animals received seven injections. At the time of sacrifice, mice were anesthetized with sodium pentobarbital (150 mg/kg, intraperitoneally) and perfused with heparinized PBS as previously described [27]. The brains were harvested immediately. The brain was split into two hemispheres; one half was flash frozen over dry ice for future biochemical studies and the other half was fixed in periodate-lysine-paraformaldehyde (PLP) for 24 h for histochemistry. Kidneys, spleen, and liver were also collected for histological assessments. After fixation, brains were placed in 2% DMSO/20% glycerol in PBS and stored until sectioning. Serial coronal brain sections (40 μm thick) were cut on a freezing microtome and placed in ethylene glycol cryoprotectant (30% sucrose, 30% ethylene glycol in 0.1 mol/l PBS) and stored at –20 °C until use.

### Mouse behavioral assessment

At the end of the treatment, mice were subjected to behavioral and sensorimotor testing. Before assessment of cognitive deficits, mice were subjected to motor testing to ensure that any treatment effects observed in the cognitive tasks could not be explained by differences in motor abilities.

### Grip strength

The forelimb and hindlimb muscle strength was determined by measuring grip strength on a grip strength meter (BIOSEB, Chaville, France). Each mouse was placed on the metal grid then gently pulled away. Grip strength is measured as peak force applied to the grid before the mouse loses grip of the mesh grid. The amount of force exerted by the mice to remain on the grid was measured in four consecutive trials. Results are shown as the average of peak tension per mouse divided by the mass of the mouse.

### Open field

Locomotor activity was measured in a square open field activity chamber measuring 40 cm × 40 cm in an open field, with the light intensity in the arena measuring between 100 and 150 lm. Prior to testing, mice were adapted to the room. Mice were then placed in the arena and allowed to roam freely for 15 min while a video camera mounted above the chamber recorded movements of each mouse. Videos were analyzed using EthoVision XT (Noldus Information Technologies, Inc.) and results were calculated based on distance traveled, mean resting and moving time (s), average velocity (cm/s), and the amount of time the animals spent in the corner or center of the arena.

### Barnes maze

We used Barnes Maze to assess hippocampal-dependent spatial learning and memory. The Barnes maze is a circular table 91.6 cm in diameter raised 33 cm off the floor and contains 20 possible escape holes on the periphery of the table. One of these holes leads to a dark escape box. For this study, sex, treatment group, one of three possible escape holes, and genotype was counterbalanced for each mouse. Mice were allowed to habituate to the room prior to any testing each day. The initial single training acquisition trial was conducted the day before trial 1. In this acquisition trial, the mouse was carefully placed under a large glass beaker at the center of the table, and gently guided to the correct escape hole in the context of spatial cues. For the remainder of testing, mice were assigned to the specific escape hole. During the testing phase, a bright light was shined directly over the table and a loud radio noise was turned on in order to motivate the animal to move toward the escape hole from the beginning of each trial until the time the mouse entered the hole. Once in the hole, the noise was turned off, and the animal was allowed to stay for 1 min in the box until being returned to its cage. The escape box and table were cleaned after each animal with 70% ethanol. Two trials were conducted per day for 5 consecutive days. After each trial, the table was rotated clockwise three holes. Trials ended when animals found the escape box or 300 s had elapsed. After the testing phase, a probe trial was conducted on the sixth day. In the probe trial, the escape box is removed and search behavior of the animals is investigated for 120 s. Latency to find the escape hole and distance traveled was recorded with a camera and analyzed using EthoVision XT.

### Novel object

The novel object recognition task is based on the natural tendency of mice to investigate a novel object instead of a familiar one. For the novel object recognition task, we used a Y-maze based on previous work in both mouse and rat models using perceptual and object recognition type experiments [40–42]. Two arms were used for this test and the third arm was permanently closed off to the animal. Arms projected from a small circular ‘neutral’ zone that did not count as an arm. At the beginning of each trial mice were placed in this neutral zone. Mice were allowed to habituate to both the room and the apparatus prior to the task. During trial 1, animals were exposed to two identical objects in the two arms for 5 min. After the first trial, mice were returned to their home cage for a delay period of approximately 90 min. During trial 2, one of the familiar objects was switched with a novel object. The data are presented as preference index, a ratio of the amount of time spent in the arm containing the novel arm over the total time spent in both arms. Objects were placed at the extreme end of each arm. The objects differed in shape, color, and height. For this study, sex, treatment group, genotype, and placement of familiar and novel objects was counterbalanced for each trial. The light intensity was approximately 100 lm. Objects and the maze were cleaned with 70% ethanol between each mouse. Trials were recorded with a camera and analyzed using EthoVision XT.

### Sandwich ELISA to measure Aβ40 and Aβ42 levels in mouse brains

Soluble levels of Aβ40 and Aβ42 were measured in mouse brain S1 fraction in a double antibody sandwich ELISA. For this ELISA, a combination of a mouse monoclonal antibody 6E10, specific to amino acid residues 1 to 16, and two different rabbit polyclonal antibodies specific for Aβ40 (R162) and Aβ42 (R165) were used as previously reported [43, 44]. OD was measured at 450 nm. The assay was performed by an investigator blinded to the group assignments.

### Statistical analysis

Statistical analyses were performed with the software GraphPad Prism 7.0 (GraphPad, San Diego, CA). Normal distribution was tested by the D’Agostino and Pearson normality test when *n* > 8 for all groups. If the data did not follow a Gaussian distribution, a nonparametric test was performed. For the Barnes maze behavioral test, a two-way repeated-measures analysis of variance (ANOVA) with Tukey multiple comparisons post-hoc test was used. For novel object recognition, a one-way ANOVA with Tukey’s multiple comparisons post-hoc test was used. Analysis of fluorescent histochemical studies of human FFPE tissue was performed using a one-way ANOVA with Tukey’s multiple comparisons post-hoc test. A two-tailed *t* test was used to analyze normalized results from immunoprecipitation studies. Immunofluorescent quantification of microglia (IBA1) immunoreactivity on mouse free floating tissue was analyzed by a one-way ANOVA with Tukey’s multiple comparisons post-hoc test. Immunofluorescent quantification of amyloid plaque (6E10/4G8) and astrocyte (GFAP) burden was analyzed by the Kruskal-Wallis test. Immunofluorescent quantification of tau (PHF1, AT8, MC1) was performed by the Mann-Whitney test. A one-way ANOVA with Tukey’s multiple comparisons post-hoc test was used to analyze soluble levels of Aβ42/40 in the female S1 fraction of brain homogenates, and Mann Whitney nonparametric tests were used to analyze levels of phosphorylated tau in the S2 fraction.

## Results

### Characterization of TWF9 in human brain tissue shows preferential specificity for AD tissue versus non-AD tissue

We previously cloned a number of IgMs that were crossreactive to multiple neurodegenerative peptides/proteins and that were shown to reverse cognitive deficits in a mouse model of AD [22, 27]. We then engineered an IgG2aκ antibody, TWF9, using the whole kappa chain and the variable region heavy chain from the parent aβComAb IgMκ GW-23B7. Western blotting under reducing and nonreducing conditions was utilized to characterize TWF9 (Fig. 1a). Fast green shows the intact IgG antibody under nonreducing conditions and the kappa light chain (~ 25 kDa) and gamma heavy chain (~ 50 kDa) under reducing conditions. Antibodies specific to kappa light chain and gamma heavy chain were used to verify these results (Fig. 1a). Since GW-23B7 had been extensively characterized by fluorescent immunohistochemistry, we initially used this to compare the staining pattern similarities between the GW-23B7 parent clone and TWF9. Since the sequences for binding specificity are identical, we hypothesized that the staining should be similar. There is almost complete colocalization between the two, suggesting that TWF9 has the same binding properties as its parent IgM antibody (Fig. 1b).

**Fig. 2:**
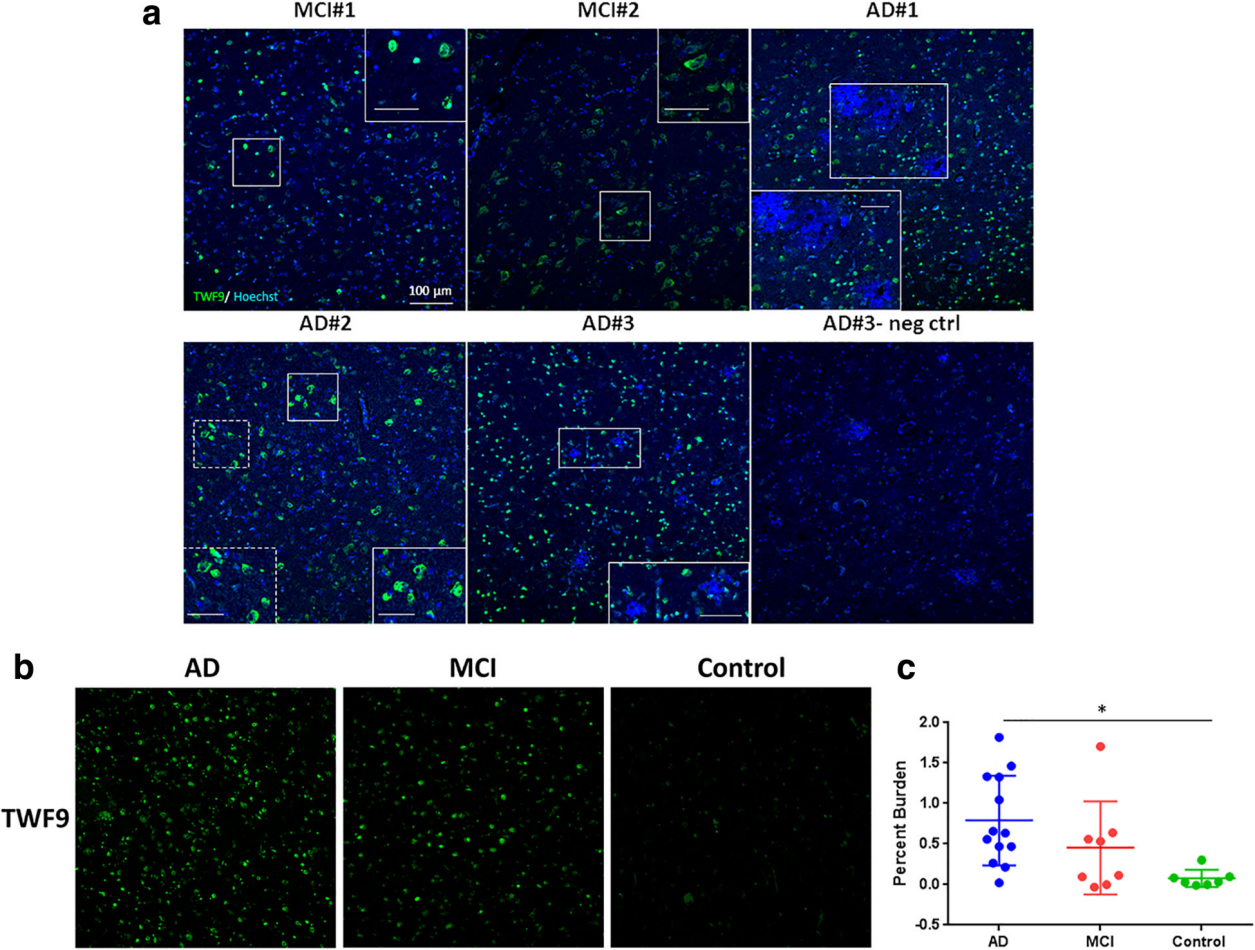
TWF9 preferentially labels neurons in AD brain tissue. **a** Alzheimer’s disease (AD) and mild cognitive impairment (MCI) cases stained with TWF9 (green) and counterstained with the nuclear marker Hoechst (blue). Immunostaining in AD and MCI tissue reveals TWF9 preferentially labels neurons. There are intercase differences in TWF9 immunoreactivity between AD and MCI cases. In AD#1 and MCI#1, TWF9 equally labels both the nucleus and cytoplasm in various neurons. TWF9 predominantly labels the nucleus in AD#3, and the cytoplasm in MCI#2 and AD#2. The scale bars located in the higher magnification inserts are 50 μm. **b** Representative images of TWF9 immunostaining in AD, MCI, and nondemented age-matched controls. **c** Quantification of fluorescent immunostaining with TWF9 in AD, MCI, and nondemented age-matched controls. Case breakdown is detailed in the Methods section. Data are expressed as mean ± SD. *p < 0.05, AD vs nondemented age-matched control by one-way ANOVA test with Tukey’s post-hoc multiple comparison analysis. neg ctrl negative control

To characterize the TWF9 antibody further, staining patterns were compared in AD and MCI brains using the hippocampal tissue sections at the level of the lateral geniculate nucleus. Initial observations revealed that TWF9 preferentially stained structures only in the gray matter. TWF9 stained within a cell structure and had relatively low background/parenchymal staining. We noted predominant cytoplasmic neuronal staining as well as nuclear staining in some neurons that was visualized by the TWF9 staining colocalizing with the nuclear stain in double fluorescent immunostaining (Fig. 2a).

To determine if TWF9 immunoreactivity was specific to AD versus non-AD tissue, we performed fluorescent immunohistochemistry in AD tissue (*n* = 13), MCI (*n* = 8), and non-AD tissue cases (*n* = 7) (Table 1). Although a few of the nondemented control cases had low staging neuropathology, there was an overall difference in TWF9 specificity among groups (*p* < 0.05; one-way ANOVA with Tukey’s multiple comparisons post-hoc test). TWF9 specificity data showed significantly higher TWF9 staining in AD cases than in nondemented control cases (*p* < 0.05; Fig. 2b, c). In contrast, there were no significant differences between AD and MCI nor nondemented controls and MCI. MCI was categorized as a GDS of 3 [30, 45]. It is important to note that in this MCI category there was a wide range of neuropathology based on the ABC score, consistent with prior studies [6, 46], and thus quite a bit of variability in TWF9 immunoreactivity. These data suggest that TWF9 is able to discriminate between AD and nondemented control tissue.

### Immunohistochemical detection of neurofibrillary tangles by TWF9

**Fig. 3:**
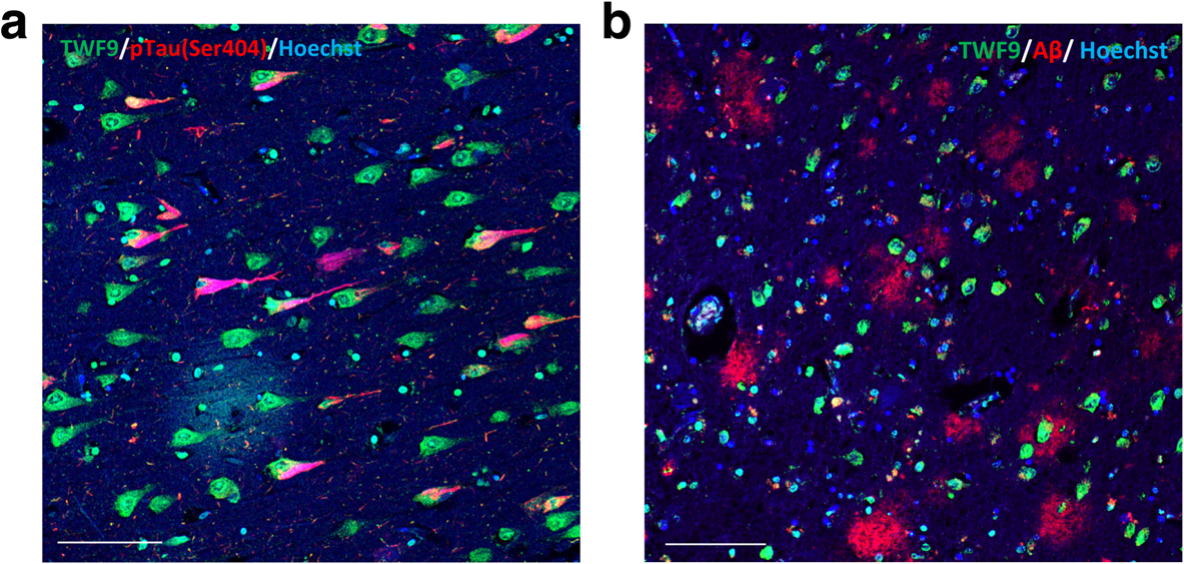
TWF9 recognizes dystrophic neurons in AD. **a** Co-staining between TWF9 (green) and AD phosphorylated tau (pTau) marker pSer404 (red) are shown in orange. **b** Co-staining of TWF9 (green) and an amyloid beta (Aβ)42-specific antibody (red) are shown. Scale bars = 100 μm

To determine if TWF9 neuronal staining was seen in neurons containing NFTs, we performed a double immunostain with TWF9 and a phosphorylated tau marker (pSer404) and saw moderate colocalization in the entorhinal cortex (Fig. 3a). This suggests that TWF9 recognizes some NFTs containing phosphorylated tau. To determine if TWF9 recognized amyloid plaques in FFPE tissue, we performed double immunostaining with TWF9 and an amyloid42 marker (rab42) in the entorhinal cortex. TWF9 did not recognize amyloid plaques or vessel amyloid deposits (Fig. 3b).

### Biochemical analysis shows TWF9 reacts with oligomeric amyloid beta and paired helical filaments (PHF)

**Fig. 4:**
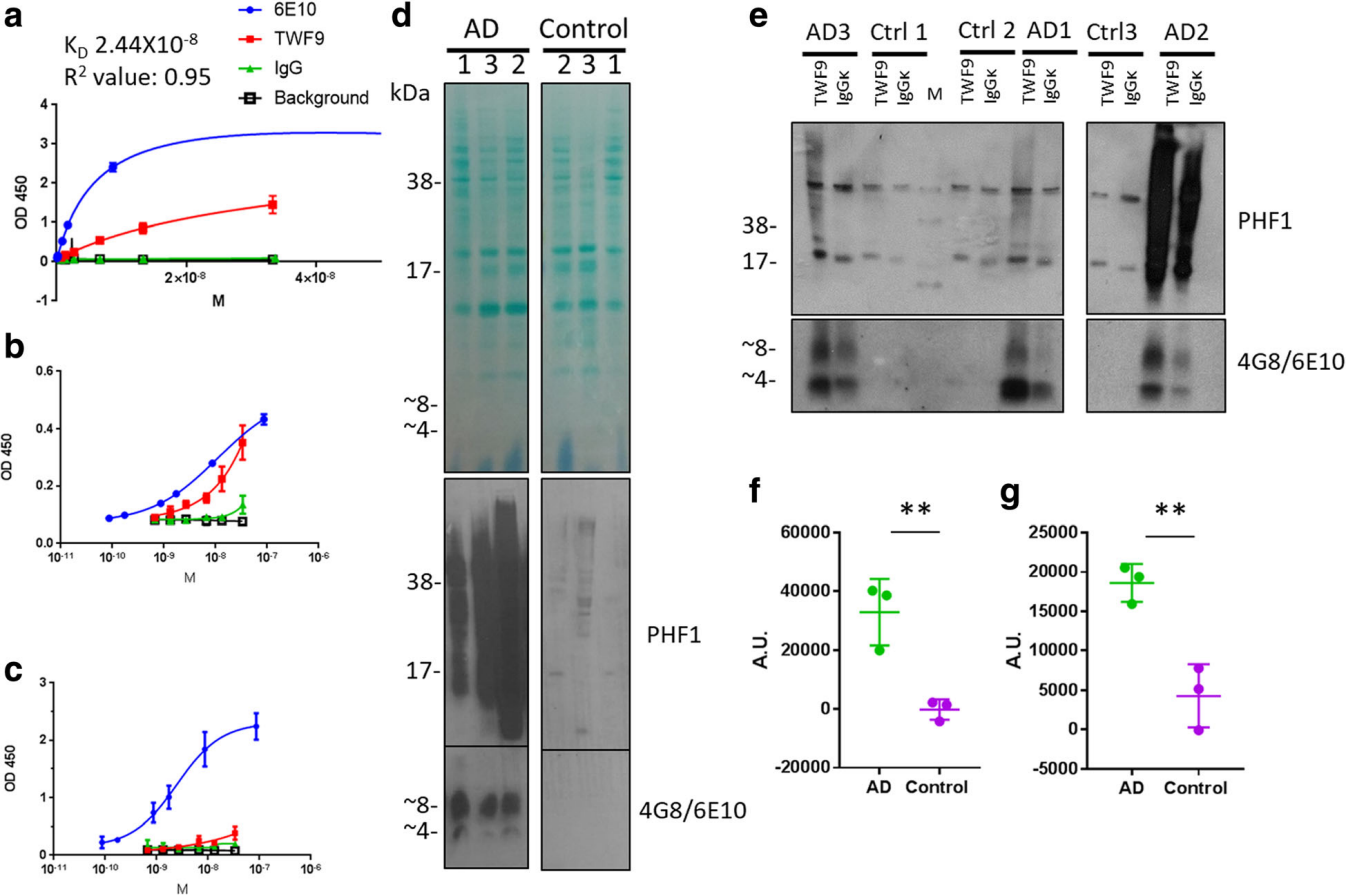
TWF9 is specific to pathological Aβ and phosphorylated tau present in AD. **a** Saturated binding curve in direct ELISA measuring binding affinity of TWF9 to Aβ oligomers. Sandwich ELISA measuring different concentrations of TWF9 and isotype control antibody affinity for **b** oligomeric Aβ42 and **c** monomeric Aβ42. Plates were coated with rabbit capture antibody specific to the C-terminus of Aβ42. **d** PHF1 antibody specific for paired helical filaments containing phosphorylated tau (pS396/pS404) and 468/6E10 antibodies specific for Aβ on an immunoblot of three Alzheimer’s disease (AD) and three nondemented age-matched control human frontal fresh frozen cortex cases used in immunoprecipitation (IP) studies. Fast green reversible protein stain is shown to assess equal loading. **e** IP product was analyzed by denaturing SDS-PAGE. Quantification of IP results for **f** Aβ (two-tailed *t* test: **p < 0.01) and **g** phosphorylated tau (two-tailed *t* test: **p < 0.01). For IP experiments, normalized values are shown as the difference between TWF9 and isotype control IgG mean gray values. Data represent the mean ± SD. A.U. arbitrary units, Ctrl control, OD optical density

To clarify the pathological proteins recognized by TWF9, we performed a variety of biochemical assays. We used a direct ELISA approach, in which Aβ oligomers were coated onto the plate, to determine the binding affinity of TWF9 for Aβ42 oligomers. Data indicate that TWF9 has an estimated K_D_ value of 2.44 × 10^−8^ M (*R*^2^ = 0.95; 95% confidence interval 1.89 × 10^−8^ to 3.227 × 10^−8^) (Fig. 4a). Next, we wanted to compare the specificity of TWF9 for oligomeric Aβ versus monomeric Aβ. Sandwich ELISAs were used in this specific set of experiments in which a rabbit antibody specific to Aβ42 C-terminus was coated onto the plate. Data indicate that TWF9 has comparable activity to that of the commercial antibody 6E10 (Fig. 4b) in binding to Aβ oligomers and does not have a strong affinity for monomeric Aβ, with activity similar to that of a nonspecific isotype control antibody (Fig. 4c). These data suggest that TWF9 is specific to the pathological β-sheet conformation present in Aβ oligomers.

Next, we performed immunoprecipitation (IP) followed by Western blot in fresh frozen AD and nondemented control brain tissue. Initial characterization of the cases used in the IP study show that the AD cases exhibited both amyloid monomer (~ 4 kDa) and dimer (~ 8 kDa) bands along with extensive phosphorylated tau as seen with 4G8/6E10 and PHF1 specific antibodies, respectively (Fig. 4d). An isotype control was run in parallel for each case analyzed by TWF9 (Fig. 4e). Immunoprecipitation shows that TWF9 significantly extracts both Aβ (Fig. 4f) (*p* < 0.01; two-tailed *t* test) and phosphorylated tau (Fig. 4g) (*p* < 0.01; two-tailed *t* test) in AD tissue versus nondemented control cases. These in vitro studies indicate that TWF9 can bind hyperphosphorylated tau and Aβ species present in the context of the brain milieu in AD.

### Passive immunization with TWF9 in old 3xTg-AD mice improves performance on cognitive tests

Previously, we have shown that chronic treatment with an anti-β-sheet conformation IgM antibody was able to improve cognition in old 3xTg-AD mice. This cognitive improvement correlated with a reduction in the soluble levels of both aggregated Aβ and soluble phosphorylated tau pathology in the soluble fraction of brain homogenates extracted from 3xTg-AD treated mice. Additionally, levels of amyloid plaques were reduced in the hippocampus and subiculum [27]. We wanted to investigate if acute treatment in 3xTg-AD mice with TWF9 could reverse memory deficits. We hypothesized that soluble species of Aβ and phosphorylated tau would be altered in the short treatment strategy without having effects on plaque and tangle pathology.

**Fig. 5:**
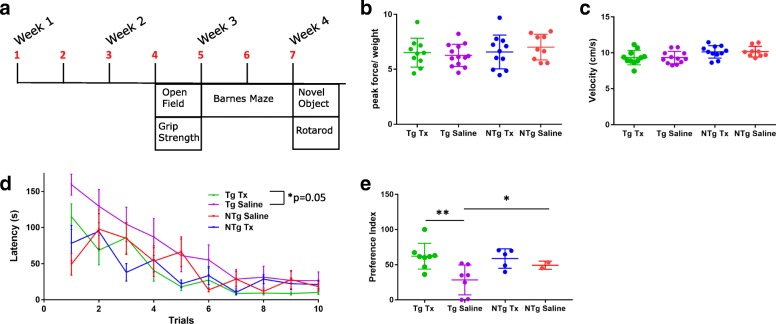
TWF9 improves performance in behavioral tests in an AD mouse model. **a** Schedule of immunization, motor, and behavioral testing. 3xTg- AD mice (18–22 months) and NTg mice (16 months) were assessed after intraperitoneal administration of 4 mg/kg (~ 100 μg per 25 g mouse) TWF9 biweekly for 2 weeks before the start of behavioral testing. Injections continued for the duration of the behavioral testing, which lasted 2 weeks. **b** Grip strength measured on all four paws of each mouse. Results are shown as the mean of four consecutive trials. Data are shown as peak force (g) exerted on grid divided by weight ± SD. **c** Average velocity (cm/s) of mice in open field arena for 15 min ± SD. **d** Spatial learning and memory testing in Barnes maze. Latency(s) to locate the escape hole is plotted. Trials ended when animals found the escape box or 300 s had elapsed. Differences between groups and effects over time were analyzed. One guided (inverted beaker) training session was conducted the day before the first trial. Two trials per day were conducted over the course of 5 days. Data shown for all animals; *n* = 11 for transgenic (Tg) treated (Tx), *n* = 12 for Tg saline, *n* = 11 for nontransgenic (NTg) Tx, *n* = 9 for NTg saline. Data are expressed as mean ± SEM. Day effect: ****p < 0.0001; treatment effect: *p < 0.05; interaction: *p < 0.05 by two-way repeated measures ANOVA followed by Tukey’s multiple comparisons test. Tg Tx vs Tg Saline (main treatment effect): *p* = 0.05. **e** Novel object recognition was tested in half the mice in two arms of a Y-maze. *n* = 7 for Tg Tx; *n* = 7 for Tg saline; *n* = 5 for NTg Tx; *n* = 2 for NTg saline. Time spent in the arm with the novel object compared to time spent in both arms is plotted as the preference index (*p < 0.05 by one-way ANOVA followed by Tukey’s multiple comparisons test). Data represent the mean ± SD. Quantification of preference index for Tg saline vs Tg Tx (**p < 0.01) and Tg saline vs NTg Tx (*p < 0.05)

Old 3xTg-AD mice (18–22 months) and old NTg (16 months) mice were assessed after intraperitoneal administration of 4 mg/kg (~ 100 μg per 25 g mouse) TWF9 biweekly for 2 weeks before the start of behavioral testing. Injections continued for the duration of the behavioral testing, which lasted 2 weeks (Fig. 5a). To determine if treatment caused any motor impairment, we first tested the animals in a battery of sensorimotor testing. Results from the grip strength test show that there were no differences between the NTg and Tg groups, nor were there differences between treated and saline-treated groups (Fig. 5b). Furthermore, no differences were observed between all four groups in velocity moved in open field arena testing (Fig. 5c).

After motor testing, mice underwent cognitive assessment. Hippocampal-dependent spatial learning and memory was assessed by Barnes maze. Latency to locate the target hole was measured. Barnes maze data show that there were significant differences in Barnes maze performance (*p* < 0.05; two-way repeated ANOVA followed by Tukey’s multiple comparisons test). There was an overall treatment effect indicating that TWF9-treated 3xTg-AD animals performed better than 3xTg-AD saline mice (*p* = 0.05) and performed comparably to the age-matched NTg groups (Fig. 5d). There was no overall significant difference between TWF9-treated and saline-treated NTg mice (Fig. 5d). Results from a single probe trial conducted on the sixth day indicate that there were no differences between treated and nontreated groups (data not shown).

A novel object recognition test was used to assess short-term memory deficits based on an animal’s exploratory behavior. This test was performed on half the mice. This novel object recognition test was modified and performed in a Y-maze based on previous protocols [40–42]. Novel object recognition data show that there are significant differences in preference index between groups (*p* < 0.05; one-way ANOVA followed by Tukey’s multiple comparisons test). TWF9-treated 3xTg-AD mice had a significantly higher preference for the novel object (*p* < 0.01) compared with saline-treated 3xTg-AD mice (Fig. 5e). Both TWF9- and saline-treated 3xTg-AD mice performed comparably to the age-matched NTg saline-treated group. NTg TWF9-treated mice had a significantly higher preference index (*p* < 0.05) for the novel object than the 3xTg-AD saline group (Fig. 5e).

### TWF9 antibody immunization leads to reduced levels of soluble levels of amyloid in female mice

**Fig. 6:**
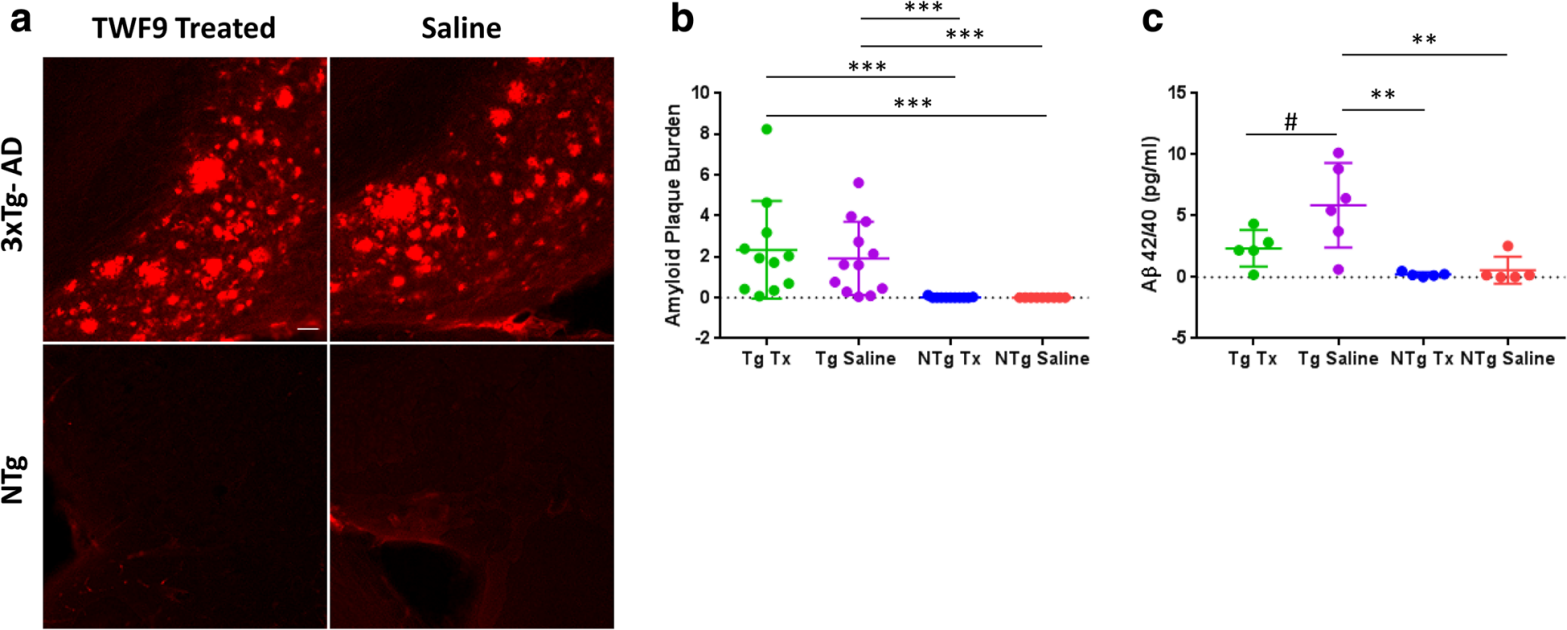
Plaque load remains unchanged by TWF9 treatment. Soluble levels of Aβ42/40 in the brain trend toward change in TWF9-treated female mice. **a** Representative images of amyloid plaque burden in subiculum of old 3xTg-AD and nontransgenic (NTg) mice. Scale bar = 100 μm. **b** Analysis of amyloid plaque burden measured as percentage of subiculum area covered by amyloid plaques. Quantification of amyloid plaques in subiculum stained with 4G8/6E10 from two to four sections per mouse (****p < 0.0001 by Kruskal-Wallis test followed by the Dunn’s multiple comparisons test); *n* = 11 for transgenic (Tg) treated (Tx), *n* = 12 for Tg saline, *n* = 11 for NTg Tx, *n* = 9 for NTg saline. Data shown as mean ± SD. NTg Tx vs Tg Tx (**p < 0.01), NTg Tx vs Tg saline (*p < 0.05), NTg saline vs Tg Tx (**p < 0.01), and NTg saline vs Tg saline (*p < 0.05). **c** The ratio of Aβ42/Aβ40 was measured in the S1 fraction of brain homogenates in female 3xTg-AD and NTg mice. Quantification of the ratio of Aβ42/Aβ40 by ELISA (**p < 0.01 by one-way ANOVA followed by Tukey’s multiple comparisons test). Results shown for females (*n* = 5 for Tg Tx, *n* = 6 for Tg saline, *n* = 5 for NTg Tx, *n* = 5 for NTg saline) as mean ± SD. Quantification of soluble levels of Aβ42/Aβ40 for Tg saline vs Tg Tx (^#^*p* = 0.0554), Tg saline vs NTg Tx (**p < 0.01), and Tg saline vs NTg saline (**p < 0.01)

We measured amyloid levels in the brains of TWF9- and saline-treated mice to determine if the reduction correlated with our behavioral results. 3xTg-AD mice are known to have high amyloid plaque burden in the subiculum; thus, we focused our analysis in this region. Since there are known significant sex-specific differences in amyloid pathology in 3xTg-AD mice [47], we performed sex-specific analysis along with analysis of overall groups. There was no difference in amyloid plaque pathology in the subiculum of the TWF9- and saline-treated 3xTg-AD mice (Fig. 6a, b), and nor were there differences when sex-specific analysis was performed (data not shown). Due to the difference in mouse and human Aβ42, it was no surprise that NTg mice did not contain amyloid plaque pathology and it is well known that nontransgenic mice do not develop plaque pathology. Thus, there were significant differences in plaque burden between the NTg and Tg groups (Fig. 6b). We also measured the levels of soluble Aβ42/Aβ40 in the soluble S1 fraction of the brain homogenates. There were no differences between groups when examining all mice together (data not shown). However, there were significant differences in a female-only subgroup analysis (*p* < 0.01; one-way ANOVA followed by Tukey’s multiple comparisons test). There was a reduction in Aβ42/Aβ40 ratios in female 3xTg-AD TWF9-treated animals (*p* = 0.06) compared with saline-treated 3xTg-AD mice (Fig. 6c). Levels of soluble Aβ42/40 were significantly lower in both NTg saline- (*p* < 0.01) and NTg TWF9-treated (*p* < 0.01) groups compared with 3xTg-AD saline-treated female mice.

### Soluble level of hyperphosphorylated tau is reduced with TWF9 passive immunization

**Fig. 7:**
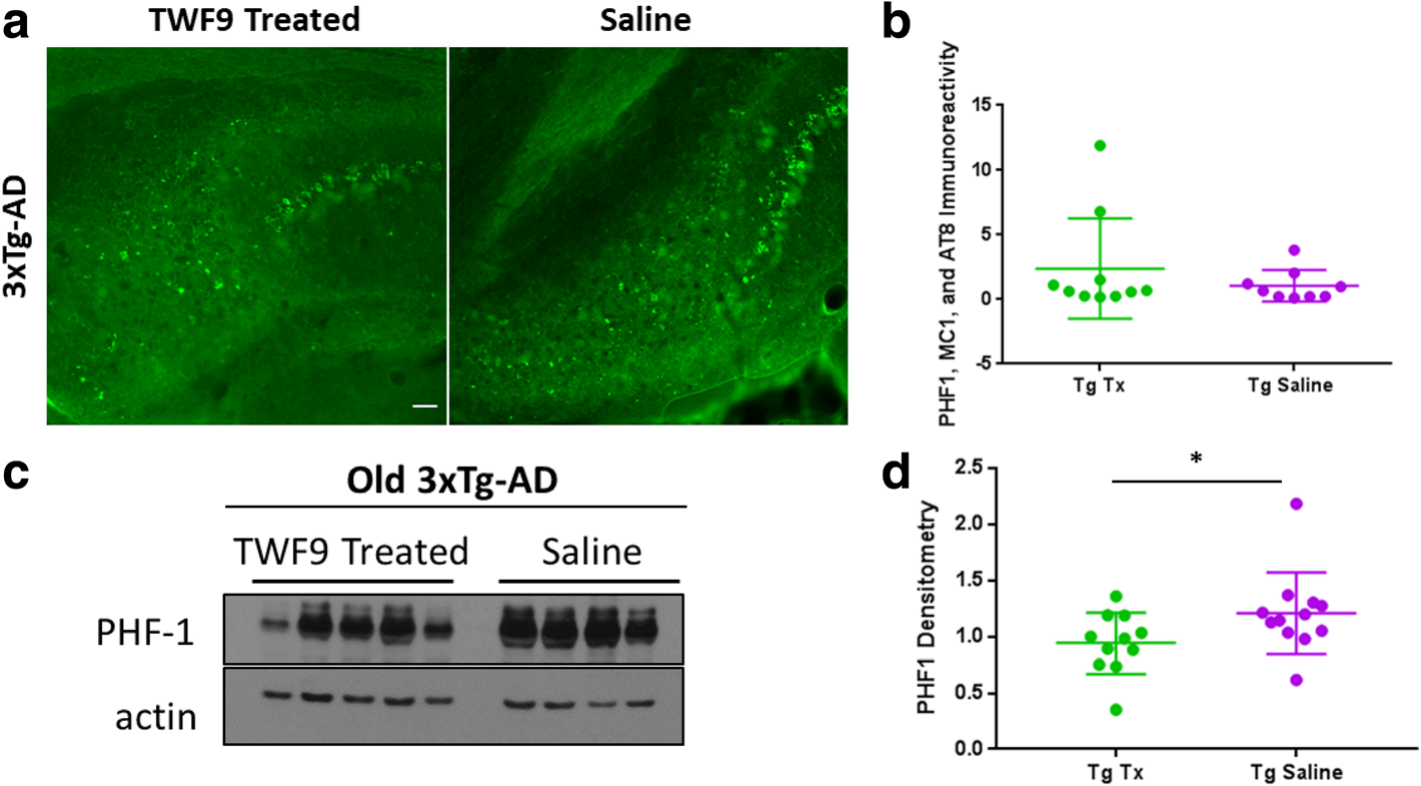
TWF9 treatment reduces soluble levels of phosphorylated tau. **a** Representative images of phosphorylated tau immunoreactivity in subiculum of old 3xTg-AD mice. Scale bar = 100 μm. **b** Analysis of tau immunoreactivity measured as percentage of subiculum area covered by phosphorylated tau. Quantification of mixed PHF1, MC1, and AT8 staining in the subiculum from two to four sections per mouse; *n* = 11 for transgenic (Tg) treated (Tx) and *n* = 12 for Tg saline. Data shown as mean ± SD. Analysis by two-tailed Mann-Whitney test. **c** Western blot analyses of tau pathology in old TWF9-treated and saline-treated 3xTg-AD mice. Abnormal hyperphosphorylated tau at tau sites (pSer396/pSer404) was measured by PHF1 in the soluble fraction of homogenized brains; *n* = 11 for Tg Tx and *n* = 12 for Tg saline. 15 μg of 3xTg-AD S2 fraction was loaded. **d** Quantification of Western blots normalized to actin is shown as mean ± SD. *p < 0.05 by two-tailed Mann-Whitney test

To determine the TWF9 immunization effects on tau pathology, we first used fluorescent histochemistry in the subiculum of 3xTg-AD mice only, since we know that NTg mice do not develop tau pathology. There were no differences in the 3xTg-AD groups (Fig. 7a, b). Then we analyzed levels of highly soluble phosphorylated tau in the S2 fraction of brain homogenates by Western blot. There was significantly less soluble phosphorylated tau in the brain homogenates of treated mice compared with controls (*p* < 0.05; Mann-Whitney test) (Fig. 7c, d).

### Immunization with TWF9 does not invoke glial activation

**Fig. 8:**
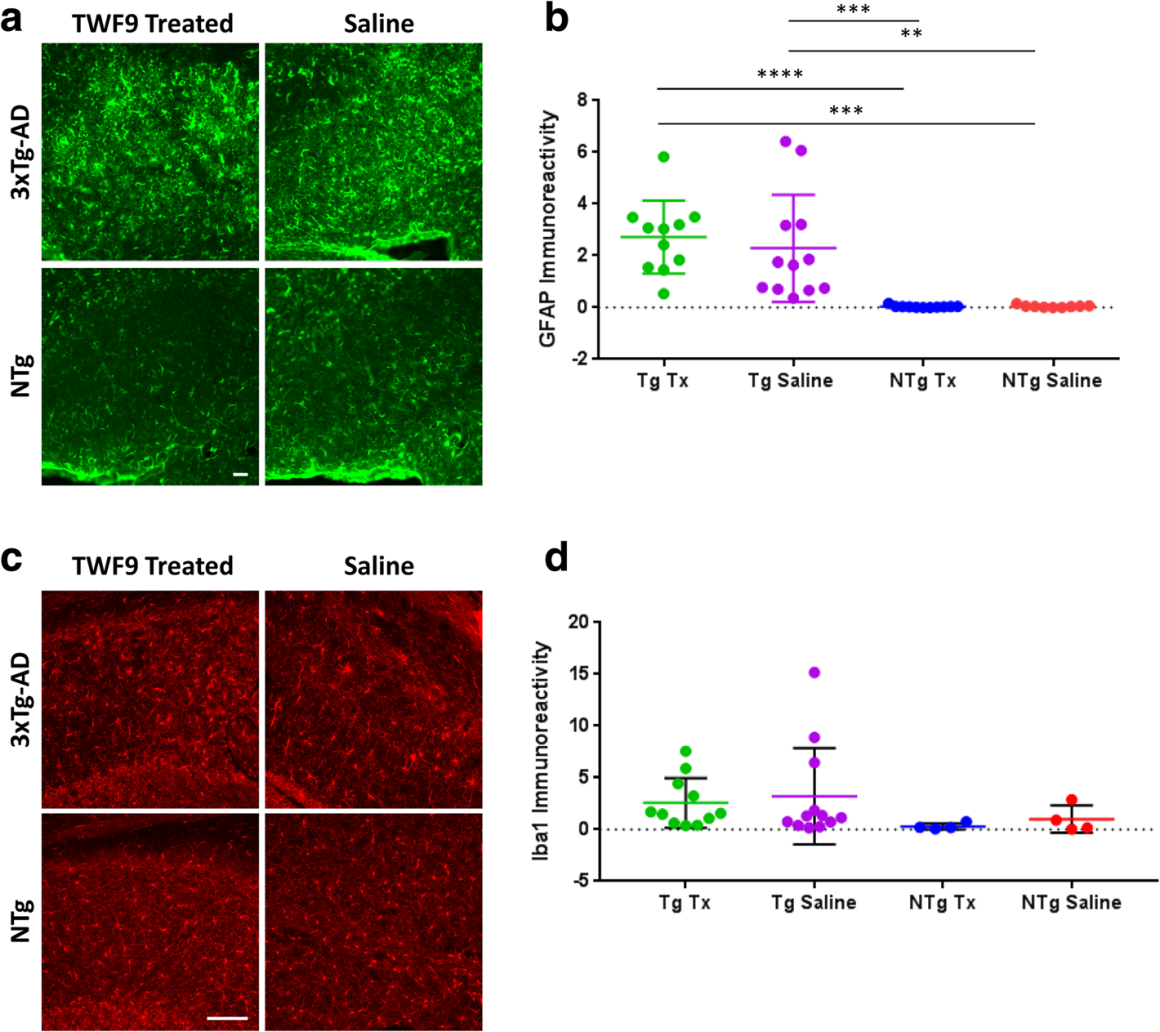
TWF9 treatment does not affect astrocyte or microglia levels. **a** Representative images of GFAP-positive astrocytes shown with GFAP immunoreactivity in subiculum of old 3xTg-AD mice. Scale bar = 100 μm. **b** Analysis of GFAP immunoreactivity measured as percentage of subiculum area covered by astrocytes. Quantification of GFAP staining in the subiculum from two to four sections per mouse of all animals (****p < 0.0001 by Kruskal-Wallis test followed by Dunn’s multiple comparisons test); *n* = 11 for transgenic (Tg) treated (Tx), *n* = 12 for Tg saline, *n* = 11 for nontransgenic (NTg) Tx, *n* = 9 for NTg saline. Data are expressed as mean ± SD. Quantification of GFAP immunoreactivity for NTg Tx vs Tg Tx (****p < 0.0001), NTg Tx vs Tg saline (***p < 0.001), NTg saline vs Tg Tx (***p < 0.001), and NTg saline vs Tg Saline (**p < 0.01). **c** Representative images of resting and active microglia shown with IBA1 immunoreactivity in subiculum of old 3xTg-AD mice. **d** Analysis of IBA1 immunoreactivity measured as percentage of subiculum area covered by microglia. Quantification of IBA1 staining in the subiculum from two to four sections per mouse; *n* = 11 for Tg Tx, *n* = 12 for Tg saline, *n* = 4 for NTg Tx, *n* = 4 for NTg saline. Data are expressed as mean ± SD. Analyzed by one-way ANOVA followed by Tukey’s multiple comparisons test

To investigate the mechanisms by which immunization with TWF9 improved performance in behavioral tasks and decreased Aβ and tau pathology, we assessed microglia and astrocyte cell involvement which is one of many possible methods by which immunotherapy exerts its effects. There was no difference in astrocyte activity associated with 3xTg-AD TWF9- and saline-treated animals; however, there were significant differences between NTg groups and 3xTg-AD groups (*p* < 0.0001; Kruskal-Wallis test followed by the Dunn’s multiple comparisons test) (Fig. 8a, b). Next, we investigated total levels of microglia in the subiculum using IBA1, which labels both active and resting microglia [48, 49]. We performed this in all Tg mice and half of the NTg mice. There was no difference between groups (Fig. 8c, d).

## Discussion

To date, there is no effective treatment for altering the disease course of AD, a disease neuropathologically characterized by fibrillar aggregates of Aβ and hyperphosphorylated tau in the form of amyloid plaques and NFTs, respectively [5–8]. Although fibrillar Aβ associated with amyloid plaques exerts toxicity, such as generating toxic reactive oxygen species when bound to copper ions, disrupting membranes, and sequestering vital components of the proteostasis network, their overall presence in the brain has poor correlation with cognitive decline [6, 46, 50–53]. Although the presence of NFTs correlates with cognitive function better than the amyloid plaque burden, this correlation remains imperfect [6, 54–56]. Therefore, although these fibrillar aggregates do cause some local toxicity, oligomeric species are thought to be the major mediators of toxicity [8, 14, 15, 27, 54, 57–59]. Oligomers have a variety of toxic activities, such as membrane perturbation, oxidative stress, endoplasmic reticulum stress, long-term potentiation inhibition, long-term depression facilitation, channel formation, and receptor dysfunction by direct receptor binding [60–65]. Importantly, oligomeric synaptic toxic effects contribute to synapse loss, and synapse loss is the major correlate of cognitive impairment in AD [51, 66–68]. Thus, oligomers may prove to be a promising therapeutic target [16, 19, 22, 27, 69].

Previous studies have shown that acute treatment with a monoclonal antibody targeting Aβ oligomers can reverse cognitive deficits without reducing the amyloid burden in treated mice [70–72]. In this present study, we treated aged 3xTg-AD mice biweekly (intraperitoneally) with 4 mg/kg TWF9 or with sterile saline for a total of 4 weeks. We chose the 3xTg mouse model since it has been widely used in AD studies and is considered amongst the most complete transgenic mouse models of AD pathology available [73, 74]. However, in the future we also plan to test TWF9 in other AD transgenic models, as well as in nonhuman primate models, since no single model mirrors all the features of human AD fully [19, 73–75]. TWF9, an anti-β-sheet conformation monoclonal antibody (aβComAb), contains the whole kappa chain and the variable region heavy chain from the parent aβComAb IgMκ GW-23B7 [27]. During treatment, animals underwent behavioral testing. We found that treated old 3xTg-AD animals performed better in the Barnes maze (Fig. 5d) and novel object recognition test (Fig. 5e), which indicated that immunotherapy with TWF9 improves performance in behavioral testing in aged 3xTg-AD mice. In this study, there was no reduction in the typical neuropathological hallmarks, amyloid plaques (Fig. 6a, b) and tau tangles (Fig. 7a, b), but rather a trend towards significant reductions in soluble amyloid beta in female 3xTg-AD TWF9-treated mice (Fig. 6c). We believe that levels of soluble amyloid beta in males were unaffected because, in this mouse model, they are known to have less amyloid beta pathology [47]. Additionally, we saw that immunization resulted in significantly lower levels of soluble phosphorylated tau (Fig. 7d). In this present study, we did not observe any toxicity associated with TWF9 immunization in aged NTg and 3xTg-AD mice.

Many promising preclinical studies have shown improved cognition in various mouse models; however, these antibodies unfortunately have failed in multiple clinical trials [16, 19, 76–78]. There have also been promising conformational antibodies developed targeting pathological Aβ and tau. These conformational antibodies are listed in our previous publication [22]. Although these antibodies have been useful in targeting either Aβ or tau oligomers, none have been able to simultaneously recognize both pathologies. This study shows the characterization of TWF9 which reacts with both Aβ oligomers and tau pathology as seen by sandwich ELISA (Fig. 4a, b) and immunoprecipitation (Fig. 4e–g). We believe that simultaneous targeting of misfolded Aβ and tau as a therapeutic strategy is likely critical to success in patients. We propose that both Aβ and tau oligomeric species need to be targeted to have effects in the presence of pre-existing extensive AD pathology. By the time an individual starts to exhibit symptoms of AD, neuropathology and other underlying pathophysiological processes are already advanced [6, 9, 79]. It is known that in patients, even at MCI or early AD stages, there is already very extensive Aβ and tau pathology [6, 9, 80]. Therefore, starting treatment in late-stage disease or even in early stages of symptomatic AD has been cited as a possible reason for the failure of a number of clinical trials [16, 81, 82]. A promising aspect of this study is the fact that treatment was performed at such a late stage of disease when amyloid and tau pathology is extensive. This increases the likelihood of a humanized version of TWF9 having effectiveness in clinical trials.

Another advantage of TWF9 is that it specifically recognizes oligomeric Aβ (Fig. 4b) and not fibrillar Aβ (Fig. 3b), associated with amyloid plaques and vessel amyloid deposits. We believe that this is important because fibrillar species are thought to be less toxic than oligomers [51, 83] and that the direct targeting of fibrillar vessel amyloid deposits is linked to the major side effect of many vaccine clinical trials, namely amyloid-related imaging abnormalities (ARIA) [23–26]. Therefore, specific targeting of the most toxic species increases the likelihood of humanized versions of TWF9s having effectiveness in clinical trials, without associated toxicity such as ARIA.

An additional potential advantage of an antibody such as TWF9 that targets abnormal β-sheet conformation is that it could be effective against concomitant pathologies such as α-synuclein oligomers and TAR DNA-binding protein 43 (TDP-43) oligomers [22, 27]. Mixed pathology is present in the majority of AD patients [46, 84–86]. Additionally, the aforementioned conformational antibodies listed in our previous publication have all been raised to self-antigens and could lead to late autoimmune toxicity. Thus, we posit that an advantage of our antibody TWF9 is that it is made to a non-self-antigen which substantially lowers the risk of adverse risks [22, 27].

It is known that antibodies can exert effects through a variety of modes of action and can increase inflammation as a result. Hence, we assessed the levels of microglial engagement in association with TWF9 treatment. Overall levels of active and resting microglia remained unchanged in all animals (Fig. 7d). Astrocyte number, morphology, and function are known to be altered during disease progression in AD [87, 88]. More specifically, astrocytes are known to take up Aβ and to be highly activated in the vicinity of amyloid plaques [88, 89]. Since TWF9 treatment did not reduce amyloid plaque burden but still had moderate effects on soluble Aβ, we wanted to determine if TWF9 treatment influenced astrocyte activity. Overall levels of active astrocytes are significantly higher in 3xTg-AD mice than NTg mice (Fig. 7b). These data suggest that reducing soluble levels of tau and Aβ oligomers is not sufficient to reduce astrocyte activity, most likely due to the advanced amyloid plaque and NFT pathology.

An additional arm of this study was to determine the effects of TWF9 immunization in an aged nontransgenic mouse strain, C57Bl/6. Other studies have shown that there are age-related changes in the behavior in C57Bl/6 mice from young adulthood to middle age [90]. We did not see any effect of TWF9 immunization on behavior in treated and saline-treated animals (Fig. 5). This could be because the behavioral changes associated with this model are not due to pathological misfolded proteins but some other underlying causes associated with normal aging [91–93].

## Conclusion

Overall, our studies suggest that acute treatment with an aβComAb, TWF9, can effectively improve performance in behavioral testing without needing to affect overall amyloid plaque and tau burden. Targeting both Aβ and tau concurrently may have a greater chance of future clinical therapeutic success in the setting of established AD pathology.

## Abbreviations

AD: Alzheimer’s disease
ARIA: Amyloid-related imaging abnormalities
Aβ: Amyloid beta
aβComAb: Anti-β-sheet conformation monoclonal antibody
ELISA: Enzyme-linked immunosorbent assay
FFPE: Formalin-fixed paraffin-embedded
GDS: Global Dementia Scale
IP: Immunoprecipitation
MCI: Mild cognitive impairment
NFT: Neurofibrillary tangle
NGS: Normal goat serum
NTg: Nontransgenic
OD: Optical density
PBS: Phosphate-buffered saline
Tg: Transgenic
THB: Tissue homogenization buffer
